# regioneR: an R/Bioconductor package for the association analysis of genomic regions based on permutation tests

**DOI:** 10.1093/bioinformatics/btv562

**Published:** 2015-09-30

**Authors:** Bernat Gel, Anna Díez-Villanueva, Eduard Serra, Marcus Buschbeck, Miguel A. Peinado, Roberto Malinverni

**Affiliations:** 1 ^1^Institute of Predictive and Personalized Medicine of Cancer (IMPPC), Campus Can Ruti, Badalona, Spain and; 2 ^2^Josep Carreras Institute for Leukaemia Research (IJC), Campus ICO-HGTP, Campus Can Ruti, Badalona, Spain

## Abstract

**Motivation:** Statistically assessing the relation between a set of genomic regions and other genomic features is a common challenging task in genomic and epigenomic analyses. Randomization based approaches implicitly take into account the complexity of the genome without the need of assuming an underlying statistical model.

**Summary:** regioneR is an R package that implements a permutation test framework specifically designed to work with genomic regions. In addition to the predefined randomization and evaluation strategies, regioneR is fully customizable allowing the use of custom strategies to adapt it to specific questions. Finally, it also implements a novel function to evaluate the local specificity of the detected association.

**Availability and implementation:** regioneR is an R package released under Artistic-2.0 License. The source code and documents are freely available through Bioconductor (http://www.bioconductor.org/packages/regioneR).

**Contact:** rmalinverni@carrerasresearch.org

## 1 Introduction

Sets of genomic regions are the output of many different genomic and epigenomic analyses like ChIP-seq peaks, regions with copy number alterations or differentially methylated regions. The identification of meaningful associations between a set of genomic regions and other genomic features is an important part of downstream analysis. The genomic features can be of different types: other region sets, intrinsic properties of the genome—e.g. GC content—or functions defined over the genome—e.g. DNA methylation levels, phylogenetic conservation. In addition, different metrics can be used to evaluate the associations. Thus, while counting the number of overlaps between two region sets is a widely used evaluation strategy, with regioneR it is possible to answer a wider range of biological questions beyond region overlapping with the use of other combinations of metrics and genomic features.

Computing any of these evaluations can be straightforward but assigning a statistical significance to the association levels is far from trivial. Due to the complexity of the genome, statistical models relying on strict assumptions can only be used in certain circumstances. Empirical approaches such as permutation tests might overcome these limitations but they require the use of the appropriate strategies to generate meaningful results and usually have higher computational costs.

Existing software tools to evaluate the association of region sets and other genomic features such as Geometricorr (Favorov *et* *al.*, 2012) or GAT (Heger *et* *al.*, 2013) are either based on statistical modelling or only applicable to a limited number of situations.

regioneR has been created to address this problem providing a fully customizable permutation test framework specifically designed to work with genomic regions. It includes a number of predefined randomization and evaluation functions covering the most frequent use cases, but the user can also provide custom functions to extend its functionality.

## 2 Features

**Fig. 1.**
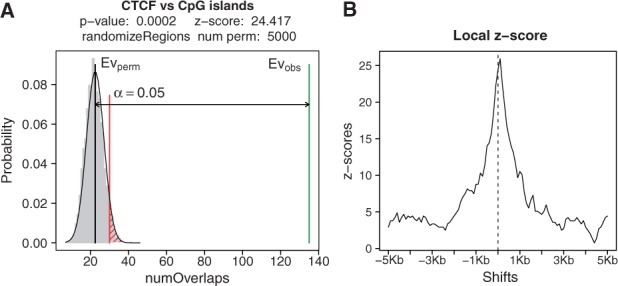
(**A**) Plot of the results of a permutation test assessing the association between a subset of 1000 HepG2 CTCF narrow peaks (ENCODE/Broad Institute) and CpG islands (Wu *et al.*, 2010), using a per chromosome randomization of CTCF peaks, the number of overlaps as the evaluation function and 5000 permutations. The association is highly significant with the observed value far from the limit of significance of the random distribution. (**B**) Plot of the local z-score of the permutation test in A. The association is strongly related to the exact position of the CTCF peaks since the z-score drops sharply as soon as the regions are shifted a few hundreds of bases

The core functionality of regioneR is a permutation test framework specifically designed to work in a genomic environment. It includes multiple randomization and evaluation strategies and the possibility to use custom functions. It can take into account the underlying structure of the genome using a genome definition and a mask. The result of performing a permutation test with regioneR is an object with the random distribution of the evaluation function, the *P* value (Phipson and Smyth, 2010) and the z-score of the test (Fig. 1A).

In addition, regionerR includes a set of helper functions based on Bioconductor’s GenomicRanges (Lawrence *et* *al.*, 2013) infrastructure to manage and manipulate region sets with a simple and consistent interface.

### 2.1 Randomization

Choosing a good randomization strategy is crucial when performing a permutation test (De *et* *al.*, 2013). The ideal strategy randomizes the regions with respect to the association being evaluated while maintaining their intrinsic structure. regioneR implements different randomization strategies: one that randomly places all regions independently along the genome with or without overlaps, one randomizing them in a per chromosome basis, a circular randomization that maintains the relative distances between the regions and a resampling strategy useful when a finite universe of possible regions is available. It is also possible to provide new randomization strategies adapted to specific problems. An expanded discussion about the built-in randomization strategies and how to create custom ones is available in the ‘Randomization Functions’ section of the package vignette.

### 2.2 Evaluation

The evaluation functions, those quantifying the association under study, are even more problem specific. A set of evaluation functions for the most common use cases has been included in the package. With them it is possible to determine either the number of overlaps or the distance between two regions sets, or to evaluate a function over the regions: methylation levels, GC content, etc. Again, it is possible to implement a custom evaluation function when needed. The ‘Evaluation Functions’ section of the package vignette includes more information about the available functions an how to create new ones.

### 2.3 Input formats

Different BED-like R objects and file formats can be used as input to any function in regioneR. The package implements a function to create a region set from different data sources: data.frame or GRanges R objects, BED or GFF files, either local or remote, and in general, any source accepted by the rtracklayer package (Lawrence *et* *al.*, 2009).

### 2.4 Genome and mask

Most functions in regioneR are prepared to work with genomes and masks, respectively, the complete set of chromosomes and the set of genomic regions ‘not available’ to work with e.g. centromeres, repetitive regions. The most suitable mask is case specific and might have an important impact on the final results. All genomes and masks provided by BSGenome (Pagès, 2012) can be used and custom genomes and masks can also be easily defined.

### 2.5 Local z-score

When performing an association analysis it is possible to detect associations that, while statistically significant, are not specific or even not biologically relevant. For example, with ChIP-seq data, it is common to detect a significant overlap between transcription factors and chromatin marks broadly distributed over gene-rich regions. This association can be true, however, it might be indirect and based on the fact that both regions tend to cluster around genes. Although we cannot decide whether an association is biologically relevant or not, with the local z-score we can at least check if the association is specifically linked to the exact position of the regions. By shifting the regions around their original position and plotting their evaluations when shifted, we can study how the value of the z-score varies. While a flat profile suggests a regional association, a sharp peak, as the one shown in Figure 1B, indicates that the association is highly dependent on the exact position of the regions. The package vignette includes a number of complete biological examples, some of which make use of the local z-score computation to further characterize the relation between two region sets.

## 3 Conclusion

regioneR provides a permutation test based framework to statistically assess the association between a set of regions and any other genomic features or annotations. It implements a number of randomization and evaluation strategies addressing the most common use cases. In addition it can also be easily adapted to specific questions by using custom functions. The package also contains a set of helper functions to simplify the management of sets of genomic regions. regioneR is a new and customizable tool to manage and analyze sets of regions, and a useful addition to the NGS and genome wide analysis toolbox.
